# Effectiveness of International Surgical Program Model to Build Local Sustainability

**DOI:** 10.1155/2012/185725

**Published:** 2012-10-22

**Authors:** William P. Magee, Haley M. Raimondi, Mark Beers, Maryanne C. Koech

**Affiliations:** Research and Outcomes Department, Operation Smile, Inc., 6435 Tidewater Drive, Norfolk, VA 23509, USA

## Abstract

*Background*. Humanitarian medical missions may be an effective way to temporarily overcome limitations and promote long-term solutions in the local health care system. Operation Smile, an international medical not-for-profit organization that provides surgery for patients with cleft lip and palate, not only provides surgery through short-term international missions but also focuses on developing local capacity. *Methods*. The history of Operation Smile was evaluated globally, and then on a local level in 3 countries: Colombia, Bolivia, and Ethiopia. Historical data was assessed by two-pronged success of (1) treating the surgical need presented by cleft patients and (2) advancing the local capacity to provide primary and ongoing care to patients. *Results*. The number of patients treated by Operation Smile has continually increased. Though it began by using only international teams to provide care, by 2012, this had shifted to 33% of patients being treated by international teams, while the other 67% received treatment from local models of care. The highest level of sustainability was achieved in Columbia, where two permanent centers have been established, followed by Bolivia and lastly Ethiopia. *Conclusions*. International missions have value because of the patients that receive surgery and the local sustainable models of care that they promote.

## 1. Background

Humanitarian medical missions often utilize international surgical teams to provide specialized surgical care to a large number of patients in a short period of time. It is an effective way to temporarily overcome limitations of the local health care system in order todeliverimmediatecare to patients in need. Despite some obvious benefits to the patients, short-term international surgical missions receive ample criticism. Some primary concerns include the failure of this model to provide sustainable solutions or ongoing access to care for appropriate medical followup. This paper examines whether the model of short-term international teams may be valuable on two different levels. It not only illustrates how this model meets a surgical need, but it examines whether this model of short-term international teams is effective in providing opportunities towards long-term, sustainable solutions in that locality and in the overall field of global health.

## 2. Introduction

Operation Smile, Inc. (OSI) is an international not-for-profit organization that specializes in treatment of patients with cleft lip and cleft palate. The organization was founded in 1982 by Bill and Kathy Magee. Since this time, Operation Smile has provided millions of patient evaluations and hundreds of thousands of free surgeries for children and young adults born with craniofacial deformities. The number of procedures performed annually by Operation Smile has grown exponentially from several hundred patients in 1985, three years after its founding, to more than eighteen thousand during the last fiscal year (2011-2012). The organization, which began in the Philippines, now has a presence in over 60 countries. 

At its inception, OSI programs were limited to short-term international missions using USA medical volunteers; however, the organization has continually concentrated on establishing local foundations to build sustainability in their countries. After receiving an invitation from a country, OSI first establishes medical missions within that country and then works to build a relationship with local medical and nonmedical volunteers. OSI aims to develop local foundations that can recruit volunteers, obtain resources, manage local missions according to local needs, and advocate with governmental and nongovernmental organizations to elevate the quality of life of cleft patients. OSI also places great importance on interacting with local business to build sustainability as evidenced in the composition of their boards—where both business professionals and physicians work together to achieve local capacity. 

After thirty years of working toward sustainability, there are now nearly 7,000 local and international specialized volunteers from over 82 countries, over half of whom volunteer locally. Globally, OSI has established 9 cleft care centers and 34 local foundations in other countries and has performed missions at 308 different local sites throughout the world. Education programs such as the Physicians Training Program, medical specialty workshops, and American Heart Association Life Support training have provided additional knowledge and skills to local volunteers, improving the care provided on and off missions. An outcomes system and evaluation process have also been developed to track patient outcomes, audit surgical performance, and provide feedback to surgeons. Formal resident and fellowship education programs have been developed thanks to the generosity of the Regan family and the Stryker Corporation. Since the founding of Operation Smile, high school and college students have been given opportunities to attend missions. There are now more than 700 students clubs in existence in the USA alone, with many more throughout the world. These students are instrumental in raising awareness and support. In the midst of this growth, international missions remain a core aspect of OSI's mission and vision as they serve as a gateway to the creation of local capacity building. 

## 3. Methods

The history and evolution of Operation Smile has been evaluated, first globally, and then on a local level in 3 countries: Colombia,Bolivia,and Ethiopia. This was accomplished by looking at historical data for the past two decades. The total number of local and international missions was calculated, and the total number of patients operated on during local and international missions was compared.

- Local missions are defined as either missions of which over 50% of the volunteers are local and/or missions of which are planned and executed by local foundations. (Missions that occur at the comprehensive cleft care centers are thus considered local missions.)
- International missions are defined as either missions of which over 50% of volunteers are international and/or missions of which are planned and executed by OSI.

Selection criteria for the three specified countries were based on the level of sustainability of the country foundation, the number of years the local foundation has been in existence, the number of locally based trained volunteers, and the number of patients annually operated on in the country. The three countries represent three different phases of self-sufficiency as determined by the number of local volunteers and by the capacity of the local foundation to plan and to execute missions effectively. 

Historical data was assessed to measure a two-pronged success of (1) treating the surgical need presented by cleft lip and palate patients and (2) advancing the local capacity to provide primary and ongoing care to cleft patients moving forward. 

The surgical need was established by approximating the number of individuals living with cleft lip and/or cleft palate at the time of the first Operation Smile program in that country. UNICEF and the World Bank provided the population estimate and the total births per year for each country. An approximation of individuals living with clefts and of the number of children born with clefts each year was then calculated for each country. Because of the lack of birth registries and of research investigating cleft incident rates, these estimates were calculated using the approximation that cleft lip and/or cleft palate affects 1 in 700 live births [1]. This established the total number of possible clefts that exist in the country. Secondly, the World Health Organization (WHO) provided the per capita total expenditure on health, the number of hospital beds, and the number of physicians for each country. These numbers were used to explain the local medical infrastructure and the countries' varying capability to establish local capacity. The number of surgeries provided by Operation Smile during five-year increments was also calculated to show how the organization has been effectively treating children with clefts. 

 The advancement of local capacity was assessed by comparing the number of Operation Smile patients cared for by international volunteers to those cared for by local nationals and how that ratio has changed over the history of Operation Smile's existence. The progression of local capacity within each of the three selected countries was assessed by documenting the establishment of local programs and systems as each foundation developed. 

## 4. Results

### 4.1. Global Analysis

**Figure 1:**
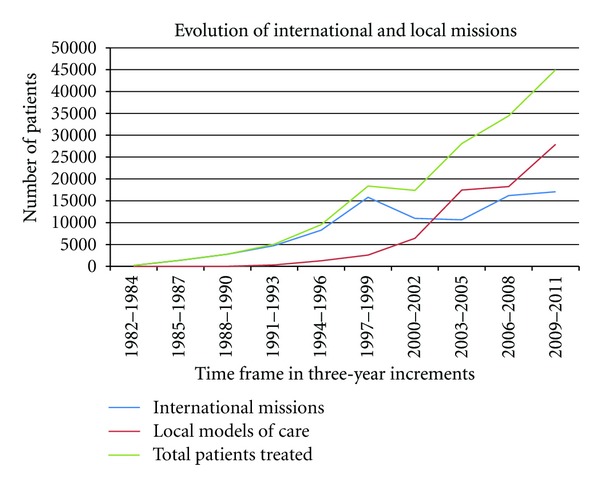
Evolution of International and Local Missions throughout History of OS.

The first measure of success was determined by looking at the number of surgeries provided since the organization's inception. The average cleft lip repair surgery can take 45 minutes to an hour to complete, and the average palate repair can last between 1 and a half to 2 hours. An international surgical mission has 5 to 6 operating tables with 1 primary surgeon and 1 anesthesia provider for each table, plus a floating surgeon and a floating anesthesia provider. This translates to an average of 150 surgeries in 5 days of surgery. While other local and international medical organizations also provide reconstructive surgery to patients with clefts, it is inevitable that developing countries will have many untreated patients. Operation Smile originally established specific sites to which they would return annually in order to reduce the prevalence of cleft lip and palate. Analyzing the historical numbers of Operation Smile missions globally, Figure 1 shows that the number of patients treated has increased consistently over time, providing one measurement of success. Figure 1 also illustrates the increase of patients on local missions versus international missions, highlighting Operation Smile's growth toward local sustainability.

### 4.2. Country Analysis

Colombia has a population of approximately 46,295,000 people, and approximately 914,000 births per year [2]. Based on the aforementioned incident rate, the country is approximated to have 6,6136 individuals living with clefts in various stages of repair. Each year, about 1,306 more children are born with clefts in Colombia. Operation Smile has operated on 14,034 of these patients during cleft missions. The country has roughly 7,198 physicians, ten hospital beds per 10,000 people, and a per capita total expenditure on health of $713 [3].

On the other hand, Bolivia, which has a population of approximately 10,090,000 and 161,440 births per year [4], has about 14,414 individuals with cleft lip and/or cleft palate and 231 newborns each year with the deformity. Of those individuals with clefts, Operation Smile has treated 2,438 patients. Approximately 10,329 physicians live in Bolivia where 11 hospitals beds exist per every 10,000 people and a per capita total expenditure on health of $233 [3].

The third country, Ethiopia, has a population of 84,730,000 and 1,779,339 births per year [5]; thus, about 121,043 individuals live with clefts, and 2,541 children are born per year with the craniofacial anomaly. Operation Smile has treated 1,466 total patients in Ethiopia. Approximately 1,806 physicians live in Ethiopia where there are 2 hospitals beds per 10,000 and $51 per capita total expenditure on health [3].

**Figure 2:**
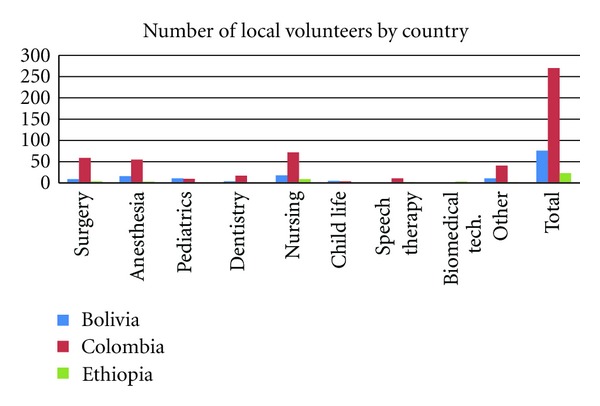
Number of local volunteers by country.

Preliminary evidence was also found to support the second measure of success,advancing the local capacity to provide primary and ongoing care for cleft patients. Over the past thirty years, OSI has shifted from 100% of patients receiving care from international teams to, in 2012, only 33% of patients being treated by visiting international teams, while the other 67% receive treatment from local models of care. The organization currently has 124 local missions scheduled for the current fiscal year 2012-2013, in comparison to 58 international missions. In the fiscal year 2008-2009, 62% of surgeries were performed during local missions [6] increasing to about 67% this past fiscal year (2011-2012).  Figure 2 illustrates the number of local volunteers that OS currently has in each of the three countries. 

The three countries that were evaluated presented three different levels of growth towards achieving local sustainability. The highest level of sustainability was achieved in Columbia, where two permanent centers have been established, and a total of 270 volunteers provide care to patients on a continuous basis. Just in the last fiscal year, 1,041 patients received free reconstructive surgery, and local programs provided over 10,000 individual and group consultations in nutrition, dental, speech, ENT, anesthesia, plastic surgery, and psychology. Operation Smile Colombia held its first local mission in 1993, and in 2002, international missions were phased out altogether. Then in 2006, OSI began holding an annual international mission with Operation Smile Colombia for the purpose of educational exchange. OSI has been active in Colombia for 24 years. 

Operation Smile has 14 years of history in Bolivia and has not reached the same level of self-sufficiency seen in Columbia. The foundation has 122 nonmedical and 23 medical volunteers, with 21 more currently in the process of being credentialed. From 2007 to 2011, OSI held surgical education modules to provide local surgeons and anesthesiologists with hands on training and mentorship. These resulted in the credentialing of 15 additional medical volunteers, enabling Operation Smile Bolivia to hold its first local mission in June, 2012. 

**Figure 3:**
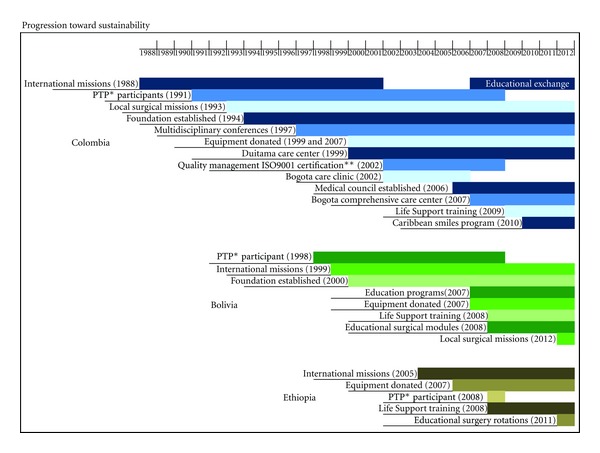
Major landmarks and progression toward sustainability. *PTP: the physicians training program involved training foundation's medical volunteers in USA hospitals. **ISO9001: International Organization for Standardization Certificate which is awarded when reaching and maintaining quality management systems requirements.

Operation Smile began in Ethiopia in 2005 and has seen very little in the way of sustainable growth. With only six locally credentialed volunteers, medical programs continue to depend almost entirely on international teams. Basic Life Support certifications and pulse oximetry training are a few of the education programs that have been offered. The most significant initiative to promote local capacity began in March 2012 with the first of many surgical education rotations, where international surgeons, anesthesiologists, and nurses will provide hands on training to local providers.  Figure 3 highlights the major landmarks and progression toward sustainability in these three countries. 

## 5. Discussion

International medical missions can serve as a valuable tool for reducing the burden of surgical disease by treating patients, while at the same time providing the opportunity to establish long-term solutions and to build local capacity. However, to build local sustainability and capacity, an organization must explore and adapt its model to the specific country's environment. Operation Smile has had to develop unique strategies to overcome various challenges which countries face. The effectiveness of a surgical program model to establish sustainability is dependent on a variety of factors, as evidenced in the following discussion of Colombia, Bolivia, and Ethiopia. 

Operation Smile Colombia has been exemplary as its growth and success continue to impact cleft patients and their communities. Having a substantially higher number of physicians and per capita total expenditure on health, Colombia has a stronger medical infrastructure than the other two countries, which has aided in its growth toward self-sustainability. Two comprehensive care centers were established in 2002 in the cities of Bogota and Duitama. Not only do the centers provide reconstructive surgery to patients, but they also offer ongoing treatment and provide resources to patients. This multidisciplinary type of care permits the centers to have the opportunity to provide specialty services to patients. Reaching such a large population would not be feasible without the local volunteer base and the establishment of the centers. In fact, local volunteers are now participating in “El Caribe Sonrie,” a project created to provide postoperative care to patients in other regions of the country. This new program, which aims to widen the impact of actions taken, increase resource management efficiency, and create local and regional autonomy in treating clefts, was locally developed and remains locally driven. Focusing on capacity building in Colombia, the program engages local health care workers and trains them in areas of psychology, speech, and oral health. The local volunteers can then provide access to rehabilitation process in social and communicational skills and adequate oral health care prevention to cleft patients. The first year of the pilot program was completed in May 2012 and has proven to be an exemplary model of how local professionals can train other locals to treat patients within their communities. OS Colombia has also witnessed the formation of other similar organizations which can be expected given the visible and inspirational nature of this type of work.

Operation Smile Bolivia was officially established in February 2000, soon after its first inaugural mission in 1999. OS Bolivia, which holds ongoing international medical programs each year, has a weaker medical infrastructure than Colombia as evidenced in the disparity between number of physicians and money spent on health care. Given the difference in total physicians in Bolivia and Colombia, it can be assumed that Bolivia has far too few plastic surgeons to meet the country's need. In 2008, the foundation began implementing regular education models which focused on training local volunteers, and thus, improving the local medical infrastructure. These modules have enabled 15 additional credentialed volunteers to join OS Bolivia's base, which is now comprised of 40 medical volunteers. The number of credentialed surgeons has also grown from one, in 2008, to three, in 2012. Additionally, the foundation now provides speech therapy through weekly workshops, monthly dental evaluations, and psychological counseling for patients and their families. OS Bolivia and OSI have worked together to provide medical professionals with Pediatric Advanced Life Support (PALS), Advanced Cardiac Life Support (ACLS), and Basic Life Support (BLS) training, in accordance with the American Heart Association's (AHA) protocols. As of June 30, 2012, 127 medical professionals have been certified in BLS, 78 in PALS, and 78 in ACLS. Because of these education initiatives, OS Bolivia has been able to grow its local volunteer base, and many local professionals have been trained in critical care. The first local mission, completed in June of 2012, marked a significant step in the history of Operation Smile Bolivia.

Since its inaugural mission in Ethiopia in December 2005, Operation Smile has conducted 16 medical missions and has provided surgery to 1,466 patients. Additionally, Operation Smile started to conduct medical education programs in various hospitals throughout the country. Over 500 Ethiopian health care professionals have completed American Heart Association (AHA) Basic Life Support (BLS) training conducted by Operation Smile. While Ethiopia has experienced some growth since its inception, the lack of infrastructure and medical personnel has impeded the organization from experiencing the local growth exhibited in countries like Colombia. This lack of medical infrastructure is evidenced in the low number of physicians, hospitals beds, and per capita income spent on health care. Because of the seriously low number of physicians in Ethiopia, the number of plastic surgeons in this country can be assumed to be much too low to treat the number of individuals with cleft. In Ethiopia, OSI aims to help build local infrastructure and also provide expertise to train local health care workers. For example, OSI has partnered with LIFEBOX, a not-for-profit organization that provides pulse oximeters and training to hospitals where this crucial monitoring tool is not currently available. OSI has developed a strong relationship with Jimma University Specialized Hospital (JUSH) and has committed to supporting the building of a new surgical center where two-week-long rotations in plastic surgery, anesthesia, and nursing will provide local health care workers with the opportunity to receive training and mentorship in small focused rotations. One such rotation, which included 49 surgeries, was completed in March, 2012. 

Operation Smile has evolved its large-scale international team mission model to one of local management and ownership. Not only has Operation Smile, in conjunction with local governments, nonprofits, and health care professionals, established nine comprehensive cleft care centers operating in seven countries, but it also has established some weekly clinics in other countries to aid in the backlog of cleft patients. The organization has treated 161,096 patients, reducing the backlog of clefts, but it has also built a solid local volunteer base with the medical knowledge, skills, and passion to continue evolving local models of care. Local foundations have engaged local businesses and hospitals to amass resources, raise awareness, and provide patient care. Involvement of international medical volunteers on missions raises awareness and establishes a link to the academic, professional, and financial resources available in more developed countries. In fact, the OSI resident programs, which promote international surgical volunteerism, have increased participants' appreciation for the burden of disease in the developing world, the participants' perspectives on global disparities regarding health care access, and the participants' “personal sense of social responsibility” [7].

## 6. Conclusion

The momentum that Operation Smile has experienced during the past 30 years has enabled the organization to impact the lives of children and communities exponentially. Building local capacity and sustainability is essential to reaching a larger population of patients. Operation Smile has created a long-standing, well-respected, effective organization whose sustainable model may aid other similar organizations in their path toward local capacity building. While the surgical interventions accomplished by short-term international teams are the most easily showcased success, in Operation Smile's history, international missions have in many cases preceded the establishment of local capacity that has a greater impact. It is the authors' belief that international missions served a substantial role in paving the way for that capacity.
